# 545. Reconstruction Using Biodegradable Temporizing Matrix for Limb Length Preservation After Below the Knee Amputations

**DOI:** 10.1093/jbcr/irag033.242

**Published:** 2026-04-06

**Authors:** Zachary F Wang, Arpana Jain, Philomene Spadafore, Erica Whetten, Karen J Richey, Kevin N Foster

**Affiliations:** Valleywise Health Medical Center, Gilbert, AZ; Diane & Bruce Halle Arizona Burn Center - Valleywise Health, Phoenix, AZ; Diane & Bruce Halle Arizona Burn Center - Valleywise Health, Phoenix, AZ; Valleywise Health Medical Center, Phoenix, AZ; Diane & Bruce Halle Arizona Burn Center - Valleywise Health, Phoenix, AZ; Diane & Bruce Halle Arizona Burn Center - Valleywise Health, Phoenix, AZ; Diane & Bruce Halle Arizona Burn Center - Valleywise Health, Phoenix, AZ

## Abstract

**Introduction:**

Below-knee amputation (BKA) involves surgical removal of the lower leg while preserving the knee joint. This procedure is indicated for irreparable lower extremity damage form burns, trauma, or necrotizing soft tissue infections (NSTI). Knee preservation is associated with improved ambulation, prosthetic fitting, proprioception, and energy expenditure. Biodegradable Temporizing Matrix (BTM), a synthetic dermal substitute, may serve as a viable option for soft tissue coverage and limb length preservation in BKA patients.

**Methods:**

The study was conducted at regional burn center and included BKA patients who had extensive soft tissue loss from thermal, traumatic, or infectious causes etiologies between 2018 and 2025. The study included patients of all ages and genders. Patients who died within one week of the BTM application were excluded. Collected data included demographics, admission diagnoses, and comorbidities. Procedural and outcome measures included BTM application time, time to split-thickness skin grafting, time to 95% healing, time to prosthetic clearance, and rates of unplanned readmissions and stump infections.

**Results:**

Eight patients were included, yielding 10 records due to two patients undergoing bilateral BKAs. The mean age was 35.7 years, with a range of 8 to 66 years. Five patients were male and three were female. Five patients were admitted for burns, and three for NSTI. The average BTM procedure time was 113.4 minutes. The mean time to grafting was 32 days, and mean time to 95% healing was 41 days. The average time to prosthetic clearance was 104 days. No unplanned readmissions occurred, and stump infection rate was 11%.

**Conclusions:**

Compared with published data on traditional stump preservation techniques, BTM may offer a cost-effective and clinically effective alternative. The observed 11% infection rate is lower than the 30–80% rates reported in prior studies. BTM appears to be a viable option for soft tissue reconstruction and limb length preservation following BKA.

**Applicability of Research to Practice:**

This study provides preliminary evidence supporting the use of BTM for limb preservation in BKA patients. Future research in comparing the outcome with alternative techniques is needed.

**Funding for the study:**

N/A.

